# Science Education in the Light of COVID-19: The Contribution of History, Philosophy and Sociology of Science

**DOI:** 10.1007/s11191-020-00143-5

**Published:** 2020-07-09

**Authors:** Michael J. Reiss

**Affiliations:** UCL Institute of Education, University College London, London, UK

**Keywords:** COVID-19, History of science, Nature of science, Philosophy of science, Sociology, Interdisciplinarity

## Abstract

In this position paper, I examine how the history, philosophy and sociology of science (HPS) can contribute to science education in the era of the COVID-19 pandemic. I discuss shortcomings in the ways that history is often used in school science, and examine how knowledge of previous pandemics might help in teaching about COVID-19. I look at the potential of issues to do with measurement in the context of COVID-19 (e.g. measurement of mortality figures) to introduce school students to issues about philosophy of science, and I show how COVID-19 has the affordance to broaden and deepen the moral philosophy that students typically meet in biology lessons. COVID-19 also provides opportunities to introduce students to sociological ways of thinking, examining data and questioning human practices. It can also enable students to see how science, economics and politics inter-relate. In the final part of the paper, I suggest that there are strong arguments in favour of an interdisciplinary approach in tackling zoonoses like COVID-19 and that there is much to be said for such interdisciplinarity in school science lessons when teaching about socio-scientific issues and issues intended to raise scientific literacy.

## Context

I am primarily a biology educator, and when considering the adequacy of school science education in a time of COVID-19, it is tempting to wring my hands and complain that when I started my school teaching career in the 1980s, we did large amounts of teaching about disease. We taught at secondary level about a whole range of human infectious diseases, with detailed life cycles showing the roles of intermediate hosts and the importance of animal-human transmission; we taught about how infectious diseases could be tackled by prevention (e.g. nets for malaria) as well as treatment and cure. We taught about the role of nutrition and general good health in reducing the likelihood of developing certain diseases and enhancing the body’s ability to respond appropriately if a person did become infected. We taught about the immune system and what happens when it fails to recognise a new pathogen or when it over-reacts. We taught about immunisation and how different approaches to it were needed for different infectious organisms. And I was at the very beginning of my teaching career when HIV/AIDS made an appearance and educators responded quite rapidly with materials and pedagogies to be used in schools (e.g. Harvey and Reiss 1987).

But there is not much use in grumbling about historical changes in educational practices, and school science education some 35 years ago did not inhabit a Golden Age. What I want to do here is to respond to Sibel Erduran’s call, as editor of *Science & Education*, for ‘Position papers about how HPS can contribute to science education in the era of the Covid-19 pandemic’ (Erduran 2020: p. 234). The paper will also have resonance to the current STEM education special issue of Science & Education. Some of the examples in the paper will illustrate that science is situated not only within history, philosophy and sociology but also it often has implicit links to mathematics, technology and engineering.

My focus is on school science education, recognising that science education takes place in a myriad of other places from those that can respond very rapidly to changing events (the news cycle on the internet, radio and TV) to those that respond more slowly (permanent exhibits in museums). My aim here is not to look at the specifics of how biology education might respond to COVID-19 but rather to examine what history, philosophy and sociology of science might contribute and the implications of this for school science. To structure my argument, I will begin by looking at these three disciplines one by one, though it will soon be evident how much they intertwine, and towards the end of this article, I will argue for the benefits of a more interdisciplinary approach to school science education.

## History of Science

In a project that is currently delayed in its pilot stage as a result of COVID-19, Catherine McCrory (forthcoming)^1^ writes about the place of history in science teaching. She points out that history too often serves in science teaching as ‘decoration’ and cites the historian of science, Hasok Chang, who, in his 2015 Wilkins-Bernal-Medawar lecture, wrote of accounts of history in science textbooks or popular media:

They tend to be ‘human interest’ stories, appearing as mere garnishes to presentations of scientific content – stories of heroic scientists who overcame adversity, tragic scientists hampered by human limitations and circumstances, fortunate scientists who made great discoveries by exploiting chance happenings, strange scientists who engaged in bizarre experiments or devised fantastical theories, and so on. (Chang 2017: p. 92)

Now, I could erect a defence of garnishes—the surface application of detail so as to delight that quintessentially distinguishes postmodernism from modernism in architecture—but instead I will follow Chang who goes on to ask whether the study of the past of science can help us improve present scientific knowledge—a key question asked in the history, philosophy and sociology of science (HPS) and addressed enthusiastically, as Chang notes, by Harvard’s Project Physics (1962–1972) and successive school curriculum initiatives. In answering his question, Chang argues that knowledge of the history of science can result in a better understanding of the scientific knowledge that is accepted at present. In addition, it can give us a better understanding of the methods that scientists use, to which I will return in the section on the philosophy of science.

Chang’s argument from the history of science is one that has had support within the science education community. Allchin, having undertaken an analysis of Mendel and genetics, Kettlewell and the peppered moth, Fleming and penicillin, Semmelweis and handwashing, and Harvey and the circulation of blood, critiqued ‘popular histories of science that romanticize scientists, inflate the drama of their discoveries, and cast scientists and the process of science in monumental proportion’ (Allchin 2003: p. 330). He concluded that ‘we do not need more history in science education. Rather, we need different types of history that convey the nature of science more effectively’ (Allchin 2003: p. 329). In an illustration of the reality that in science education, we often seem to reinvent rather than build on previous findings and arguments, Milne had earlier critiqued ‘heroic science stories’, pointing out that ‘science stories transmit both knowledge and values’ (Milne 1998: p. 186).

Chang only mentions ‘motivation’ once in his article—and then rather negatively in his final paragraph where he writes ‘I noted that history is often used in order to excite curiosity and give inspiration for science, and that this motivation often encourages distortions and oversimplifications of history’ (Chang 2017: p. 104). However, as McCrory (forthcoming) points out, student motivation matters. When I used to teach secondary students, I peppered (a form of garnish) my lessons with accounts of the lives and work of the scientists behind the science that the students were learning. There were, no doubt, plenty of occasions when even a school history teacher, let alone an academic historian of science, might have cringed on hearing me, but the function of such teaching was not so much for me to teach my students about the history of science, it was to engage them, to motivate them. Only occasionally—the role of Mendel, Darwin and Wallace in the theory of evolution is a notable example—were the historical stories key to the science.

When we focus on COVID-19, it seems clear that history has lessons that can help students both the better to understand the emerging science and to appreciate how science is undertaken. Some of the aims of this teaching will depend on the circumstances under which the teaching takes place. I am writing this in early May 2020 where the widespread presumption in many countries is that we are over the worst of the pandemic and what is needed now is a roadmap to restoring countries to normality, so that people can get back to work and to normal social interactions. Much school teaching, in so far as it is taking place, is occurring on-line or via other modes of distance learning. The reality is that for a biology teacher, this absence of face-to-face contact makes it more difficult to discern and take account of how students are feeling—it may, be, for example, that some students are scared, others grieving, others bored.

The most obvious way that a biology educator might see the role of history of science in a time of COVID-19 is by considering past pandemics. Few students will know that the infectious disease that has killed the most humans over the last two centuries (records before that time are poor in quality) is tuberculosis (TB), caused by the bacterium *Mycobacterium tuberculosis* (Paulson 2013). To this day, over a million people a year die from it (1.5 million in 2018—the latest year for which good-quality data have been published) (World Health Organization 2020a). Remarkably, about a quarter of all people around the globe have latent TB—but they do not develop symptoms unless their immune system become severely compromised, for instance through HIV infection or because of malnourishment resulting from something like homelessness.

TB is spread primarily by the inhalation of tiny water droplets with the bacteria that are released when someone who has pulmonary or laryngeal tuberculosis coughs, sneezes, laughs, shouts, etc. This transmission route is also one that COVID-19 has. However, unlike COVID-19, TB is not spread via contact with infected surfaces—touching does not spread TB unless the bacterium is breathed in. A closely related disease, bovine TB, is caused by *Mycobacterium bovis* and spread from cattle to other mammals, including humans. As with most topics in science, the history of TB is fascinating, and a host of factors—pasteurisation of cow’s milk, improved living standards and general health, the development and increasing use after the Second World War of the Bacillus Calmette–Guérin (BCG) vaccine—has led to it being less of a problem in wealthy countries (Lienhardt et al. 2012). The involvement of cattle in the spread of TB has similarities with the importance of animal-human transmission for COVID-19, and there is on-going controversy as to the relevance of badgers in bovine TB (TB in cattle) and about how bovine TB might best be tackled (McCulloch and Reiss 2017).

Personally, I would garnish the tuberculosis story with a sprinkling of the terrifying roll call of those who have died from TB: just from the world of literature, there are Anne and Emily Brontë, Elizabeth Barrett Browning, Anton Chekhov, Franz Kafka, John Keats and George Orwell, who survived long enough to be treated in 1948 with the antibiotic streptomycin (discovered in 1943), before dying in 1950.

**Fig. 1:**
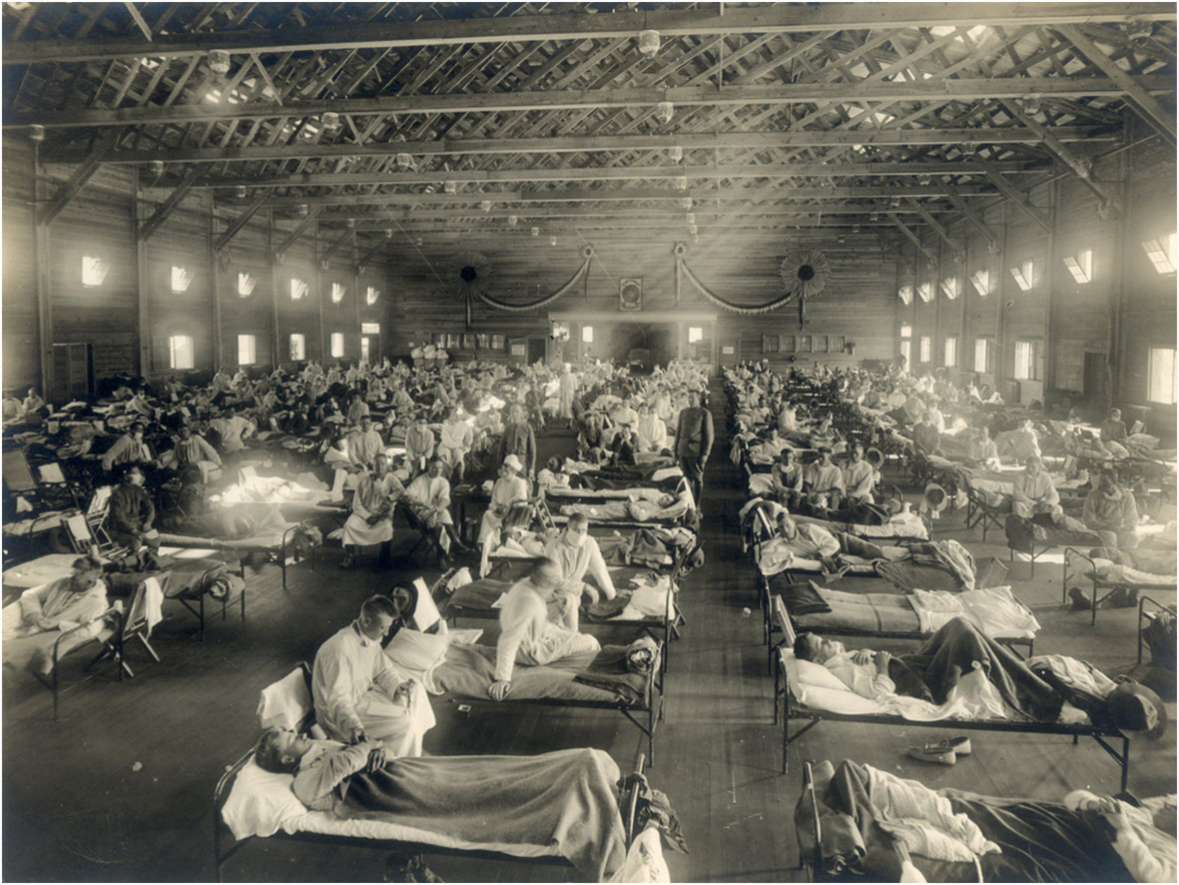
Camp Funston, at Fort Riley, Kansas, during the 1918 influenza pandemic. Taken from https://upload.wikimedia.org/wikipedia/commons/b/bc/Camp_Funston%2C_at_Fort_Riley%2C_Kansas%2C_during_the_1918_Spanish_flu_pandemic.jpg

The pandemic that is most often mentioned in the context of COVID-19 is the 1918–1919 influenza pandemic (see also the 1976–1977 swine flu epidemic in the USA (Neustadt and Fineberg 1978)). It has been estimated that about 500 million people became infected with the influenza virus (one-third of the then world’s population) and about 50 million people died (a mortality rate of about 10%). Like COVID-19, the disease was another example of a zoonosis (a disease transmitted to humans from non-human animals), being caused by an H1N1 virus with genes of avian origin (Jordan et al. 2019), but, unlike COVID-19, mortality seems to have been highest in people younger than 5 years old, 20–40 years old and 65 years and older (Fig. 1).

It is not known where the 1918–1919 influenza pandemic originated—though it was probably in the USA, Europe or China (Taubenberger 2006). The disease is often referred to as ‘Spanish flu’. The reason for this is not that it originated there but that Spain was one of the few European countries to be neutral in the First World War. Wartime censors in other countries suppressed the news of the influenza, fearing its adverse effect on morale. It is often the case that countries name diseases after other countries, in an attempt to deflect blame from those in power and to stigmatise foreigners:

Syphilis had a variety of names, usually people naming it after an enemy or a country they thought responsible for it. The French called it the ‘Neapolitan disease’, the ‘disease of Naples’ or the ‘Spanish disease’, and later *grande verole* or *grosse verole*, the ‘great pox’, the English and Italians called it the ‘French disease’, the ‘Gallic disease’, the ‘*morbus Gallicus*’, or the ‘French pox’, the Germans called it the ‘French evil’, the Scottish called it the ‘*grandgore*’, the Russians called it the ‘Polish disease’, the Polish and the Persians called it the ‘Turkish disease’, the Turkish called it the ‘Christian disease’, the Tahitians called it the ‘British disease’, in India it was called the ‘Portuguese disease’, in Japan it was called the ‘Chinese pox’, and there are some references to it being called the ‘Persian fire’. (Frith 2012: p. 50)

There are interesting parallels with COVID-19, which Donald Trump, of course, has more than once referred to as ‘the Chinese virus’. Less well known is the story behind the World Health Organization calling the virus ‘the COVID-19 virus’. Viruses are named by the International Committee on Taxonomy of Viruses (ICTV) who have named the causative agent for COVID-19 ‘severe acute respiratory syndrome coronavirus 2’ (SARS-CoV-2). However, as the WHO explains:

From a risk communications perspective, using the name SARS can have unintended consequences in terms of creating unnecessary fear for some populations, especially in Asia which was worst affected by the SARS outbreak in 2003.

For that reason and others, WHO has begun referring to the virus as “the virus responsible for COVID-19” or “the COVID-19 virus” when communicating with the public. (World Health Organization 2020b)

**Fig. 2:**
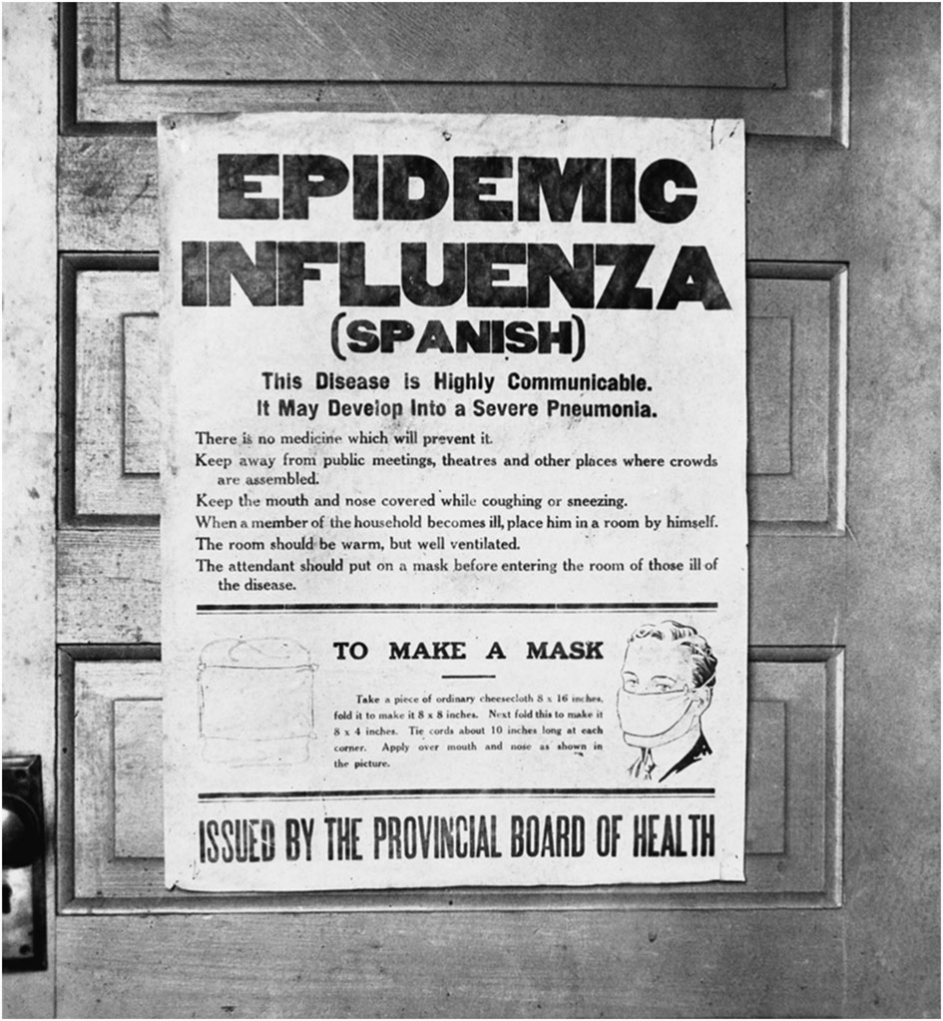
1918 influenza epidemic poster issued by the Board of Health in Alberta, Canada. Taken from https://upload.wikimedia.org/wikipedia/commons/thumb/6/61/SpanishFluPosterAlberta.png/946px-SpanishFluPosterAlberta.png

Finally, there are similarities between current attempts to tackle COVID-19 and historical attempts to tackle the 1918–1919 influenza pandemic (Fig. 2). Masks were used, public gatherings banned, schools and businesses closed, good hygiene practices recommended, makeshift hospitals established and desperate (unsuccessful) attempts made to manufacture a vaccine. In the end, it was herd immunity that caused the disease to die out. If it is herd immunity that causes COVID-19 to die out, we will have lost millions of people.

## Philosophy of Science

There is much overlap between the history of science and the philosophy of science and there is, of course, an enormous literature on the nature of science (NOS). In my own country, England, we have long favoured a simplified version of Popperian science in our accounts for school students as to how scientific knowledge is built up. As I have written previously:

Popper’s ideas easily give rise to a view of science in which scientific knowledge steadily accumulates over time as new theories are proposed and new data collected to discriminate between conflicting theories. Much school experimentation in science is Popperian in essence: we see a rainbow and hypothesise that white light is split up into light of different colours as it is refracted through a transparent medium (water droplets). We test this by attempting to refract white light through a glass prism, we find the same colours of the rainbow are produced and our hypothesis is confirmed. Until some new evidence causes it to be falsified, we accept it. (Reiss 2007: 63)

This is not the place to give a 101 account of the philosophy of science. More profitable, I think, is to look at how some of the core issues to do with the philosophy of science might usefully be addressed when teaching at school level about COVID-19. We can start with perhaps the most basic thing students are taught to do when beginning to study science—namely to measure carefully, whether they are determining the length of an object, its mass its temperature or whatever. Let us consider the measurement of mortality that results from COVID-19.

We can start by noting that it is very likely that countries under-report deaths from COVID-19. Some of the reasons for this are overtly political but others are to do with more fundamental issues to do with scientific measurement. For a start, attributing cause of death is often a matter of judgement even if we possess perfect knowledge about the circumstances of a person’s death. Consider someone who, under the influence of alcohol, falls and hits their head on a kerb and so dies. Was their death caused by the kerb, the alcohol, the breakup of their relationship that caused them to drink too much, their parents’ poor marriage, which failed to provide a model for a successful relationship or what? One thinks of Aristotle’s material, formal, efficient and final causes and of Hume’s writing about the inherent difficulties of discerning causes.

For school students, they could think about why it is difficult to determine whether people have died as a result of COVID-19. Reasons, in addition to the more general issues raised in the preceding paragraph, include the fact that many people die without a clear-cut diagnosis of COVID-19, in part due in the large majority of countries to a lack of capacity with testing (a consideration which leads to the underestimation of mortality resulting from COVID-19). Students could also be helped to realise that just because I die and am shown by testing to have COVID-19 does not necessarily mean that I died because of COVID-19 infection—as per the above fact that about a quarter of those across the globe who die would test positive for TB but the vast majority of such individuals do not die because of TS infection (a consideration which leads to the overestimation of mortality as a result of COVID-19).

Then, there are what might be termed the indirect consequences of COVID-19 on mortality. To list just some of these, fewer people go to hospital for treatments because they are afraid of becoming infected with COVID-19 there (leading to an increase in mortality rates); greater anxiety and other mental health issues with outcomes that include suicide (leading to an increase in mortality rates); an increase in domestic violence (leading to an increase in mortality rates); lower levels of exercise and increased food consumption (possibly leading to an increase in mortality rates); lower levels of traffic (leading to a decrease in mortality rates); lower levels of air pollution (leading to a decrease in mortality rates); and so on. The point of this litany is not for students to learn it off by heart but to think about the indirect effects that COVID-19 might have on mortality.

In schools, students are all too often given the impression that measurement is a trivial issue—something that with a bit of an effort and some care that they should be able to sort out straightforwardly. Measurement relies on mathematics knowledge, and it can be considered a cross-cutting theme in STEM related problems. At best, they are taught something about random and systematic errors and anomalous results. In reality, careful measurement lies at the heart of science and raises a number of philosophical issues (Tal 2017). For a lovely account of what the boiling point of water is, as determined by measurements of it (spoiler alert—water only boils at its official boiling point under very distinctive circumstances), see Chang (2008)—and the issue is often as much to do with what to measure as to how to measure it.

With regard to what to measure, while mortality is what makes headlines, healthcare decisions are rarely made on mortality alone. Students might be introduced to at least two complicating factors. The first is that it may not be that health systems attempt to minimise (or should attempt to minimise) mortality but to maximise what are called QALYs (quality-adjusted life years). The second complicating factor, to which I return below, is to do with the economics of health care rationing.

QALYs are an attempt to deal with the obvious truth that most people do not so much want to live longer per se as to have more years of good health. QALY calculations therefore attempt to combine the additional years of life that are expected to be gained from a successful intervention with a measure of the effects for patients on the quality of their lives. Everyone accepts that actually measuring QALYs is an inexact science but it is generally thought to be better than not trying to. One QALY equals 1 year in perfect health, so that an additional year of life has a maximum QALY of 1 and a minimum of 0 (or even, some maintain, less than 0 if the quality of one’s life is such that one would be better off dead). Measuring the additional years of life from a successful medical intervention can be estimated with some confidence; measuring the quality of life after a medical intervention is much more difficult, and there are various methods used of which the most common is the entirely subjective one of asking people to rate their quality of life on a scale from 0 (I would be as well off if I were dead) to 100 (perfect).

The relevance of QALYs to COVID-19 is that while we are still in the early phase of the pandemic, it is clear that many of those who recover from COVID-19 will have a reduced level of quality of life—for example, because they will require life-long renal dialysis or a kidney transplant. State medical systems have, when resources are finite, to make decisions about how much to treat people and QALYs are used to help facilitate such decisions. If something like QALYs are not used, health systems can end up spending all their resources on keeping a relatively small number of people alive when many others could be treated or enabled never to become ill in the first place (e.g. through public health initiatives) for the same amount of financial investment and medical time (also often a limiting resource).

In the above, I have focused on the measurement of mortality and issues to do with the quality of life, but there are other important issues to do with COVID-19 and measurement. In particular, the value of the basic reproduction number (R_0_), i.e. the average number of new infections generated by an infectious person in a totally naïve population, and of the subsequent reproduction rate (R), i.e. the average number of new infections generated by an infectious person at any time, are both difficult to determine. Estimates of R_0_ for COVID-19 currently vary by a factor of more than two (Liu et al. 2020), and there is inevitably a time lag between the human behaviours that affect R and subsequent measurements of it. Students can also be helped to appreciate that R is affected by a very large number of variables to do with both the person who is already infected and those whom they may go on to affect (including, age, gender, population density, presence of underlying health conditions and a number of variables to do with behaviour, such as extent of social interactions and personal hygiene). Students might also be helped to appreciate that TB, while it has a similar value of R to that of COVID-19 (a recent review gave values that range from 0.24 to 4.3 (Ma et al. 2018)), is far less contagious, in the sense that it is substantially less likely that a person with TB will spread it to someone else per unit of time that they spend in each other’s company. TB, unlike COVID-19, influenza or colds, usually only spreads between family members who live in the same house.

I have concentrated in this section on issues to do with measurement. When measurement is considered in undergraduate physical science courses, the emphasis is on issues to do with quantum theory. As is well known, Heisenberg’s uncertainty principle states that there is a fundamental limit to the precision with which certain pairs of physical constants can be measured (iconically, momentum and position). At the appropriate stage of their education, students can be helped to enquire whether this is a constraint that results from the effect of observers or whether it is a constraint that is inherent within all wave-like systems. However, there are, as indicated above, many other issues to do with measurement that can help students appreciate how the rigorous thinking and conceptual clarity that (should) characterise philosophical thinking, including thinking about the philosophy of science, can help illuminate issues of central relevance to COVID-19.

Some of these issues are considered in school biology courses, for example, statistical issues to do with sampling (resulting from limitations on access to data), but others are less often considered with any degree of explicitness, for example, the importance of biological objects being historical products (Montévil 2019). Of course, measurement is only one issue with which the philosophy of science concerns itself. But I hope that I have shown that there is plenty here to profitably occupy school students when learning about COVID-19 issues.

## Moral Philosophy

Moral philosophy can obviously be considered as sitting within philosophy but I have given the discipline its own section here, in part because even at school level, ethics is often given a certain prominence in biology.

As is the case with debates about the place of history in science education, there is a long-running debate about the place of ethics in science education (Reiss 1999). Objections to the inclusion of ethics in science education include the claim that ethics simply does not fit there on epistemological grounds (any more, for example, than aesthetics does) and that science teachers lack the expertise to teach it. Those in favour of including ethics in school science can point to the fact that mathematics is epistemologically distinct from science but we include plenty of mathematics in science and that many students find that the inclusion of ethics in science makes the subject more engaging and ‘relevant’ for them. There is also the argument that including ethics can lead to a better understanding of science (understood narrowly); for example, discussing ethical objections to in vitro fertilisation or cloning can lead to a deeper examination of questions about when human life begins and what we understand by individuality.

There are a number of ethical issues raised by COVID-19 that would make for good discussion in the classroom. I will mention two here: health care rationing and vaccination.

Health care rationing is not often discussed in school biology lessons, where the focus is more often on the acceptability of new technologies, such as genetic engineering or cloning, environmental issues, such as pollution and the loss of biodiversity as a result of human activities, and (sometimes) beginning and end of life issues. The reality, though, is that health care rationing nearly always exists, even if it is often kept hidden and even though, in countries with public health care systems, people like to presume that it does not exist. With COVID-19, the initial threat in the spring of 2020 that health care systems around the world would be overwhelmed led to a more explicit acknowledgement of health care rationing as there were near panics about the availability of ventilators and other items of equipment, not to mention the availability of doctors, nurses and other health care professionals. The ethical issues that flow from such shortages are principally to do with who gets privileged access. For example, should someone in the prime of life with young children be favoured over someone in poorer health in their 80s with no dependent relatives?

At the time of writing, we do not know whether it will be possible to develop one or more vaccines against COVID-19. Unlike health care rationing, vaccination is often covered in school biology, though the coverage can focus simply on the science with vaccination being presented as an unproblematic success story (Reiss 2018). After an account of Edward Jenner’s classic 1796 experiment on 8-year-old Edward Phipps (itself more than a little ethically problematic by today’s standards), graphs are presented showing dramatic decreases, thanks to vaccination, in the incidence of such diseases as smallpox and polio.

However, objections to vaccination began almost as soon as the practice was introduced. Nineteenth century objections included arguments that they did not work and were unsafe (Ernst and Jacobs 2012) or that their compulsory introduction (e.g. the 1853 Compulsory Vaccination Act in the UK) violated personal liberties (Durbach 2000). To this day, vaccination is rejected by some for much the same reasons. Such individuals are often castigated by health care experts and portrayed as selfish. I have nothing against passionate teaching and learning but in a school setting, there is the opportunity to examine more carefully the arguments for and against vaccination in a way that can be difficult in students’ homes. Such ethical examination goes hand-in-hand with mainstream science teaching—for example, teaching that if herd immunity is to help prevent the spread of a disease through vaccination, a certain percentage of the population (the percentage varies inversely with R_0_) needs to have acquired immunity.

Done poorly, ethics teaching can become no more than a list of arguments for and against certain practices. To enhance the quality of students’ arguments, students need to be introduced to some of the main ethical frameworks—most likely consequentialism, duties and rights, and virtue ethics. My own view is that it is easier for science teachers to teach about ethics than it is for specialist ethics teachers to teach about science, which is another reason for including ethics in the science classroom rather than hoping that the ethical implications of science get covered somewhere else in the school curriculum.

However, if ethics is to be covered in school science, students need to be assessed appropriately, both formatively and summatively (Reiss 2009). When one looks at examples of the summative assessment of ethics in school philosophy and religious studies courses, one finds that good candidates are expected to be able to write at some length and to craft a developing argument. Furthermore, banding, rather than the allocation of precise marking points, is often employed in the mark schemes used by the organisations that set the official school examinations in philosophy and religious studies. Notable too is the expectation that candidates taking these examinations should be able to criticise major ethicists and be familiar with the contrasting views of a range of both classical (e.g. Kant, Sartre, Bentham) and contemporary (e.g. Singer) authors. At present, such features are rare to the point of non-existence when ethics is examined at the end of school science courses.

## Sociology

Within HPS, the contribution of sociology is probably best known through the work of T. S. Kuhn and the subsequent science wars. More generally, sociology is the discipline principally concerned with how people behave in society. The specific field of medical sociology traditionally analysed such things as patient-doctor relationships but has grown to encompass any of the cultural (as opposed to biological) effects of medical practice. In relation to COVID-19, medical sociologists (indeed, sociologists more generally) are therefore interested in such things as who gains access and who does not gain access to technologies for prevention and treatment. Classically, much sociology looked at the importance of social class, gender and ethnicity on matters like living conditions, work patterns and wages. It is already clear that all three of social class, gender and ethnicity, along with disability, are of great relevance for the likelihood of someone becoming infected by COVID-19 and dying as a result. There is therefore a normative element to medical sociology, which therefore overlaps in its interests with moral philosophy.

There is now the growing emergence of a body of specialised sociological analysis in relation to COVID-19. Sadati et al. (2020) point out that one of the most important consequences of the COVID-19 outbreak has been the worldwide creation of social anxiety. They link this to Ulrich Beck’s pioneering book *Risk Society* (Beck 1992), in which Beck (as did other sociologists such as Giddens) argued that while societies have always been exposed to risks, modern industrialised societies are particularly exposed to risks that are the result of modernisation itself. Indeed, it is clear that contemporary practices in food production and travel have at the very least fuelled the COVID-19 pandemic. Brown (2020) also hones in on issues to do with risk, pointing out that they cannot be equated with probabilities, and draws on Mary Douglas’ classic work on everyday rituals and their purpose and her assertion that ‘it is essential for each culture to believe that the other cultures cherish wrong-headed concepts of justice’ (Douglas 2007: p. 9).

It is not, of course, my contention that school students should be introduced in science lessons to the work of sociologists like Beck and Douglas. Rather, students can be introduced, in the context of COVID-19, to sociological ways of thinking and ways of examining data and questioning human practices. Such activities can help students the better to appreciate, for example, the enormous differences between countries in terms of how they have reacted to the pandemic—denial, lockdown, social distancing, use of masks, use of technologies for contact tracing, faith in a vaccine or treatments, etc.

I have already commented how sociology overlaps with moral philosophy. It also overlaps with disciplines like politics. In my final section, before some conclusions, I examine the contributions of disciplines other than history, philosophy and sociology to science education, as exemplified by COVID-19, though I recognise that some sociologists would include much of economics and politics within their own discipline.

## Other Disciplines

In schools, students are often given the impression that scientific knowledge comes first (in terms of temporality) and is then applied to technological problems. The economics behind science, the politics of the societies within which science is funded and enacted and issues to do with psychology are rarely considered. Yet, these disciplines have great influence on the science that is undertaken and then used in society. Ziman (2000) describes ‘the Legend’ as being that account of science that is entirely realist and keeps it separate from such influences.

**Fig. 3:**
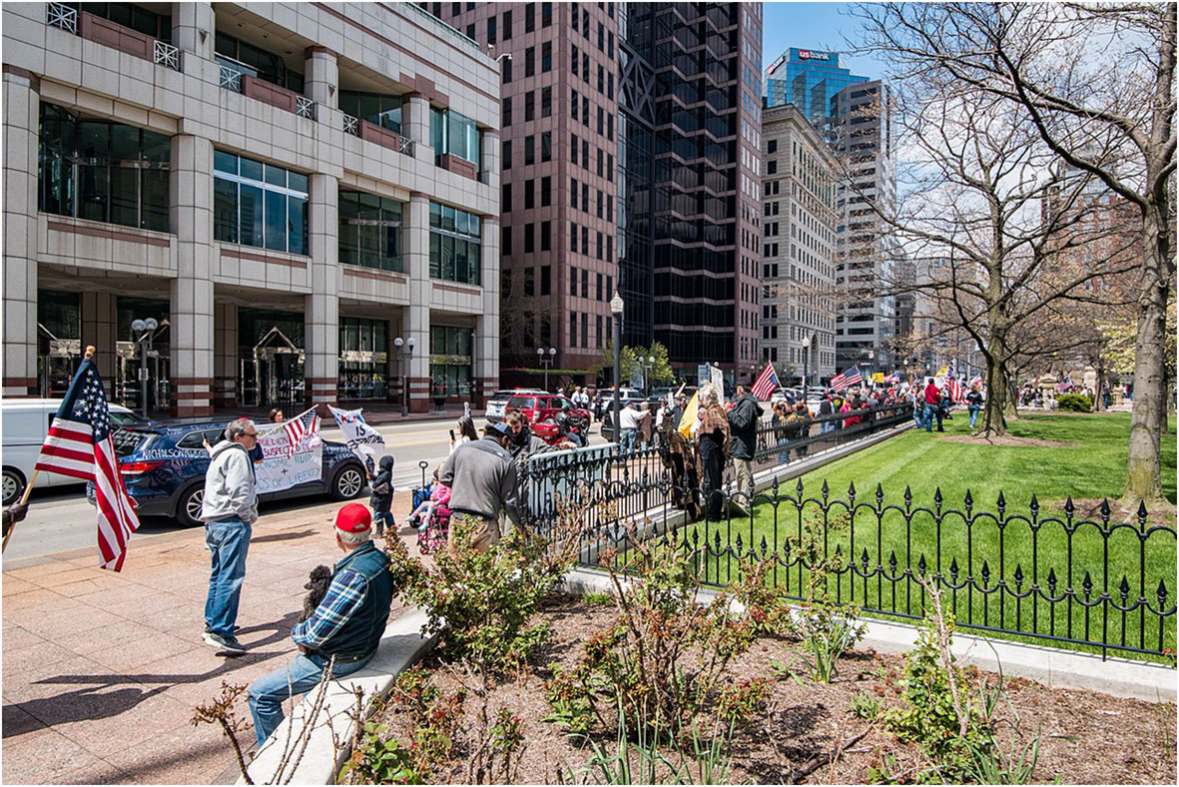
Columbus COVID-19 protests at the Ohio Statehouse, USA, 18 April 2020. Taken from https://upload.wikimedia.org/wikipedia/commons/thumb/5/5e/Columbus_coronavirus_protests_at_the_Ohio_Statehouse%2C_2020-04-18a.jpg/1280px-Columbus_coronavirus_protests_at_the_Ohio_Statehouse%2C_2020-04-18a.jpg

Few school students are likely to need much persuading of the relevance of economics and politics to COVID-19. In terms of politics, COVID-19 provides an opportunity for students to consider how democratic and non-democratic governments (including those in monarchies, theocracies and totalitarian regimes) can differ in their response to events. The importance of psychology is perhaps not quite as clear—though one could try asking students why COVID-19 has caused far more draconian governmental action than other viruses that are either more dangerous (e.g. Ebola, SARS) or regularly infect huge numbers of people (e.g. the various influenzas) or to diseases such as tuberculosis (mentioned above) that, at the time of writing, kill many more people every year than COVID-19 has to date. Students can also reflect on the diversity of opinions within countries about attempt to contain the virus (Fig. 3) and the psychological factors that might be behind these.

The mention of economics, politics and psychology raises the more general issues of interdisciplinarity. Back in 2013, Melissa Leach and Ian Scoones published an article with the title ‘The social and political lives of zoonotic disease models: Narratives, science and policy’, the abstract of which is worth citing in its entirety:

Zoonotic diseases currently pose both major health threats and complex scientific and policy challenges, to which modelling is increasingly called to respond. In this article we argue that the challenges are best met by combining multiple models and modelling approaches that elucidate the various epidemiological, ecological and social processes at work. These models should not be understood as neutral science informing policy in a linear manner, but as having social and political lives: social, cultural and political norms and values that shape their development and which they carry and project. We develop and illustrate this argument in relation to the cases of H5N1 avian influenza and Ebola, exploring for each the range of modelling approaches deployed and the ways they have been co-constructed with a particular politics of policy. Addressing the complex, uncertain dynamics of zoonotic disease requires such social and political lives to be made explicit in approaches that aim at triangulation rather than integration, and plural and conditional rather than singular forms of policy advice. (Leach and Scoones 2013: p. 10)

Leach and Scoones provide a powerful argument for the benefit of an interdisciplinary approach in tackling zoonoses like COVID-19.

## Conclusions

Science curricula, pedagogies and assessment should not be changed in a knee-jerk reaction whenever some new science-related issue arises. Nevertheless, science curricula, perhaps especially biology ones, have a history of changing appropriately in response to important science-related issues that arise in society. It seems likely that COVID-19 constitutes such an instance.

The question then arises, for those convinced, as I am, of the value of HPS for science education, as to how HPS can usefully play a role. I have tried to sketch out some possibilities above. Of course, not everyone is convinced of the worth of HPS in school science. In such circumstances, and focusing on COVID-19, though the point holds more generally, those of us keen to see HPS playing more of a role in science education might profitably argue that the history of science, the philosophy of science and the sociology of science can all help promote scientific literacy and the public understanding of biology (cf. Reiss et al. 2020).

COVID-19 can also be presented as a socio-scientific issue. In a recent article, Hancock et al. (2019) examine how science teachers collaboratively design SSI-based curricula. They note that SSI curriculum design requires careful consideration of the focal SSI to ensure that it features both social and scientific components. They found that issue selection by teachers was characterised by iterative discussion in the three dimensions of leveraging existing resources, mobilising passions and exploring issue relevance. At present, COVID-19 resources are beginning to be developed and it is certainly a topic that is relevant and likely to mobilise passions.

## Footnotes

1 The project is titled BRaSSS (Broadening Secondary School Science) and is funded by Templeton World Charities Foundation as part of their Big Questions in Classrooms programme https://www.templetonworldcharity.org/our-priorities/big-questions-classrooms.
